# Fretfulness in Infancy

**Published:** 1907-11-02

**Authors:** William P. S. Branson

**Affiliations:** Assistant Physician, East London Hospital for Children.


### Treatment.

### FRETFULNESS IN INFANCY.

By WILLIAM P. S. BRANSON, M.D., M.R.C.P., Assistant Physician, East London Hospital for Children.

I propose to deal with the detection and treatment of some of the less obvious disorders of infancy which find a common expression in fretfulness and apparently causeless irritability. The upper limit of age to which the following remarks apply is variable, being set by the age at which a child is able to indicate the site of its pain or the cause of its discomfort. This, of course, is subject to wide variation. I have no present concern with infants who are manifestly ill, but rather with those of tolerably healthy aspect who nevertheless make life a burden to those about them by constant screaming.

There are four causes of such fretfulness: Pain, hunger, ill-temper—ranging from mere discontent to a sense of real injury—and alarm. These latter emotional states are seldom of long duration, but deserve passing mention, especially the variants of ill-temper. It is rash to postulate much about infant psychology, but there is no doubt that infants form habits, both bad and good, with great facility. There is a well-defined type of the fretful baby which is evolved by mothers who, in an excess of zeal, dandle their infants, and very likely nurse them at the breast, whenever they cry. Such children are quick to learn their power, and soon become inveterate screamers. Of a similar type are infants accustomed to an artificial teat or "comforter." Babies habituated to these instruments will scream at the loss of them, and, as they are liable to fall from the mouth fifty times a day, such infants spend their time in a rapid succession of tantrums. There is no remedy for these conditions except a cessation of random nursing and the rejection of the "comforter." The bad habit is easily broken, but a day or two of riot must be anticipated.

I pass from these almost frivolous but real causes of fretfulness to deal briefly with the more common ones which give rise to pain. In this direction the digestive system is the prime offender, and of its disorders those most frequently operative are constipation and a mild grade of gastro-enteritis. In the case of breast-fed infants the combination of habitual fretfulness with a healthy aspect of body almost invariably signifies constipation and, one may suppose, associated colic. Certainly a regular relief of the bowels will cure the fretfulness in a large proportion of these cases, apart from any other interference. The cause is generally a deficiency of fat in the breast-milk, a deficiency which may be made good by securing to the mother a more generous diet, especially in the direction of proteids and fats, such as milk, meat, and eggs. Should this plan fail, an alkaline mixture of olive or cod-liver oil, in doses of four or five minims, given immediately before each feed, will usually affect a cure.

Speaking more at large, one may say that constipation is the explanation of frequent screaming in a large number of instances, both among breast-fed and hand-fed infants. If digestion, as evidenced by the colour of the stools, appears to be satisfactory, the following principles alone or in combination, may be applied with a good prospect of success.

1. To secure a larger intestinal residue by increasing the bulk of the diet. This may be done by giving, once or twice a day, half an egg beaten up in milk, or by the occasional addition of a little malted food. I am strongly of opinion that unconverted starch should not enter the menu of a child in its first year.

2. To increase the fat of the diet, either by the addition of cream, or by giving half a drachm of olive oil twice a day, or by means of the oily mixture mentioned above.

3. By inculcating a good habit, that is, by placing the infant on the chamber at fixed hours.

The milder forms of gastro-enteritis with which we are concerned evidence themselves in motions of an unhealthy colour and consistency. Diarrhœa is not by any means an essential associate of this disorder, and in consequence the anomaly may easily be neglected by the mother in giving her information unless a direct question is put as to the nature of the stools. The commonest variants are on the one hand loose grey or greenish motions containing undigested curd, and on the other clay-coloured pasty ones. In either case such motions are exceedingly offensive. They suggest a disturbance of the small intestine, and may endure for weeks. A satisfactory method of dealing with this state of things is to be found in the following procedure. The child is to be kept in bed, and particular attention paid to the temperature of the extremities. These patients usually suffer from coldness of the hands and feet, a continuance of which condition militates strongly against cure. The hands and feet should therefore be wrapped in cotton-wool, and washing reduced to the barest necessities of cleanliness. An initial dose of castor oil is followed by starvation for 24 hours. This should be absolute as regards food, but water should be given freely at the regular hours of feeding. At the expiry of this interval feeding is resumed with milk largely diluted, say four or five times, with lime-water, the proportion of milk being subsequently very gradually increased. For a week a powder such as the following should be given on alternate nights. Grey powder 1 grain, powdered rhubarb 3 grains. Such a line of treatment is commonly effectual even in out-patient practice. Before concluding this section of the subject it is worth while to mention, as a practical hint, that a warm application, such as a linseed poultice, to the belly of a peevish infant is almost unfailing as a temporary remedy for infantile fretfulness.

There are one or two conditions occasionally productive of fretfulness which at the same time are easily overlooked unless they are suspected. The commonest of these is otitis media. This has often no other expression than attacks of screaming, and should always be considered as a possible cause of the symptom. The examination of the membrana tympani should, in my opinion, be conducted under an anæsthetic. The risk is minimal—for a very slight degree of anæsthesia is sufficient—and is fully justified by the gain of ease in the examination. If the membrane is bulging, it should be punctured with all necessary precautions. If it be merely inflamed, a mixture containing a minim of the tincture of opium should be given four-hourly for a day or two, and fomentations applied to the ear.

Lastly, we have to consider two lesions whose occurrence is infrequent, but whose early detection is of supreme importance—namely scurvy and caries of the spine. One of the earliest and most striking features of infantile scurvy is extreme tenderness. Such babies will scream wildly whenever they anticipate movement, yet, in the milder grades of the disease, have a general appearance of well-being. It must be remembered that spongy gums and periosteal hæmorrhages are symptoms of the advanced disease, and that tenderness may be present without them. Infantile scurvy is a disorder of children of the better classes—children whose diet is carefully chosen and rigidly restricted to sterilised milk and patent foods. Apparently causeless tenderness in such children may often be cured by relaxation of rigidity in the diet, and by the administration of a little fruit juice. Tuberculous caries of the spine occasionally occurs in well-nourished infants. Its first symptom is screaming, due to pain on movement.

